# Risk of pneumocystosis after early discontinuation of prophylaxis among HIV-infected patients receiving highly active antiretroviral therapy

**DOI:** 10.1186/1471-2334-10-126

**Published:** 2010-05-21

**Authors:** Chien-Yu Cheng, Mao-Yuan Chen, Szu-Min Hsieh, Wang-Huei Sheng, Hsin-Yun Sun, Yi-Chun Lo, Wen-Chun Liu, Chien-Ching Hung

**Affiliations:** 1 Department of Internal Medicine, National Taiwan University Hospital and National Taiwan University College of Medicine, 7 Chung-Shan South Road, Taipei, Taiwan 100; 2 Taiwan Centers for Disease Control, 6, Linsen South Road, Taipei, Taiwan 100

## Abstract

**Background:**

Risk of pneumocystosis after discontinuation of primary or secondary prophylaxis among HIV-infected patients before CD4 counts increase to ≧200 cells/μL (early discontinuation) after receiving highly active antiretroviral therapy (HAART) is rarely investigated.

**Methods:**

Medical records of 660 HIV-infected patients with baseline CD4 counts <200 cells/μL who sought HIV care and received HAART at a university hospital in Taiwan between 1 April, 1997 and 30 September, 2007 were reviewed to assess the incidence rate of pneumocystosis after discontinuation of prophylaxis for pneumocystosis.

**Results:**

The incidence rate of pneumocystosis after HAART was 2.81 per 100 person-years among 521 patients who did not initiate prophylaxis or had early discontinuation of prophylaxis, which was significantly higher than the incidence rate of 0.45 per 100 person-years among 139 patients who continued prophylaxis until CD4 counts increased to ≧200 cells/μL (adjusted risk ratio, 5.32; 95% confidence interval, 1.18, 23.94). Among the 215 patients who had early discontinuation of prophylaxis after achievement of undetectable plasma HIV RNA load, the incidence rate of pneumocystosis was reduced to 0.31 per 100 person-years, which was similar to that of the patients who continued prophylaxis until CD4 counts increased to ≧200 cells/μL (adjusted risk ratio, 0.63; 95% confidence interval, 0.03, 14.89).

**Conclusions:**

Compared with the risk of pneumocystosis among patients who continued prophylaxis until CD4 counts increased to ≧200 cells/μL after HAART, the risk was significantly higher among patients who discontinued prophylaxis when CD4 counts remained <200 cells/μL, while the risk could be reduced among patients who achieved undetectable plasma HIV RNA load after HAART.

## Background

With the widespread use of highly active antiretroviral therapy (HAART) after 1996, HIV-related mortality and the risks of several major opportunistic infections, such as *Pneumocystis jirovecii *pneumonia (formerly *P. carinii *pneumonia), disseminated *Mycobacterium avium *complex infection, and cytomegalovirus diseases, have significantly declined in patients receiving HAART [1-3]. For example, an incidence rate of 20 cases of pneumocystosis per 100 person-months was reported in the pre-HAART era [4], which decreased to 1.2 cases per 100 person-years (100 PY) of follow-up in the era of antimicrobial prophylaxis for pneumocystosis and HAART [5]. In the HAART era, the majority of cases of pneumocystosis occur in patients who are unaware of their HIV infection or have delay in seeking HIV care, or in those with advanced immunosuppression (CD4+ count < 100 cells/μL) who have limited access to HAART and related HIV care [6,7].

In the guidelines recommended by the National Institutes of Health, the Centers for Disease Control and Prevention, and the HIV Medicine Association of the Infectious Diseases Society of America [8], HIV-infected patients should receive trimethoprim-sulfamethoxazole (TMP-SMX) as primary prophylaxis for pneumocystosis if they have CD4 counts of <200 cells/μL. Primary prophylaxis for pneumocystosis can be discontinued in HIV-infected patients who respond favorably to HAART with an increase in CD4 counts to >200 cells/μL for >3 months. For those who develop pneumocystosis, secondary prophylaxis can be discontinued when their CD4 counts increase from < 200 cells/μL to ≧200 cells/μL for more than 3 months as a result of HAART. Several observational studies and clinical trials have shown that the risk of pneumocystosis is low when primary or secondary prophylaxis is discontinued after CD4 count increases to ≧200 cells/μL. In a meta-analysis of 14 published studies, the incidence rate of pneumocystosis was estimated 0.24 per 100 PY and 0.20 per 100 PY in patients who discontinued primary and secondary prophylaxis, respectively, after CD4 counts increased to ≧200 cells/μL [9].

Prophylaxis or treatment with TMP-SMX is not without problem, however. Skin rashes and fever are the most common adverse effects of TMP-SMX in HIV-infected patients in addition to neutropenia, gastrointestinal intolerance or Stevens-Johnson syndrome [10]. Due to adverse effects of TMP-SMX and limited availability of the alternative agents for prophylaxis, such as pyrimethamine, dapsone, atovaquone, and pentamidine, a certain proportion of the patients that are in need of primary or secondary prophylaxis according to the guidelines may have to discontinue prophylaxis before their CD4 counts increase to ≧200 cells/μL. However, studies to assess the safety of early discontinuation of prophylaxis for pneumocystosis before the CD4 counts increase to ≧200 cells/μL have not been performed until recently when D'Egidio and colleagues followed 19 HIV-infected patients with sustained HIV RNA levels < 50 copies/ml and a median of CD4 count of 120 cells/μL who discontinued secondary prophylaxis without relapse after an observation duration of 13.7 months [11]. Though limited by a small sample size and a short observation duration, this study suggests that discontinuation of prophylaxis for pneumocystosis may be safe in selected patients who continue to receive HAART despite of low CD4 counts.

In this study, we aimed to assess the risk of pneumocystosis among HIV-infected patients with baseline CD4 counts of <200 cells/μL who discontinued primary or secondary prophylaxis before their CD4 counts increased to ≧200 cells/μL after receipt of HAART.

## Methods

### Study population

Between 1 April, 1997 and 30 September, 2007, consecutive HIV-infected patients who sought HIV care at the National Taiwan University Hospital were enrolled in an observational study to assess the impact of HAART on the incidences of AIDS-defining opportunistic illnesses. The study protocol has been described before [12,13]. In brief, a standardized case record form was used to record patient characteristics, baseline and sequential CD4 counts and plasma HIV RNA load (PVL), antimicrobial prophylaxis or treatment, antiretroviral therapy, occurrence of AIDS-defining opportunistic illnesses at baseline and during follow-up, pneumonia or other bacterial infections, and parasitic infections.

**Figure 1:**
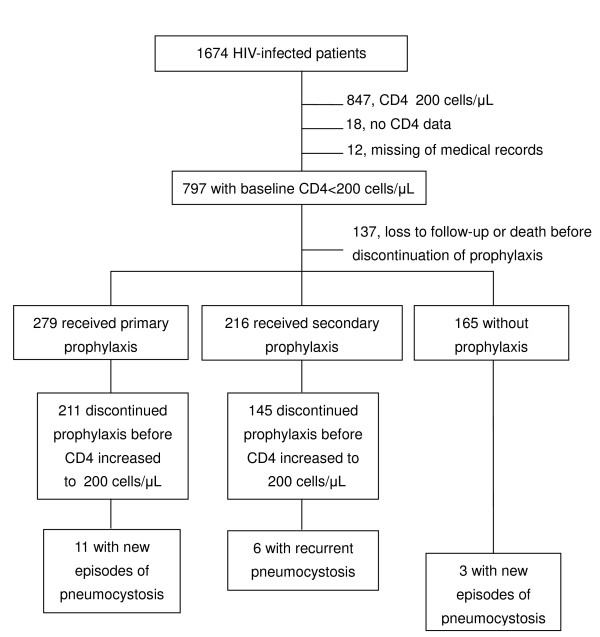
**Study population of *Pneumocystis jirovecii *pneumonia after discontinuation of primary or secondary prophylaxis between April 1997 and December 2007**.

To assess the impact of HAART on the incidence rate of pneumocystosis, we identified patients who had baseline CD4 counts of <200 cells/μL and had been followed up for more than 3 months. Patients who were lost to follow-up or died within 3 months of enrollment were excluded (Figure 1).

### Prophylaxis for pneumocystosis and HAART

In Taiwan, HIV-infected patients are provided free-of-charge access to HIV care, including HAART that was introduced on 1 April, 1997, at several designated hospitals around Taiwan. HAART consists of two nucleoside reverse-transcriptase inhibitors plus non-nucleoside reverse-transcriptase inhibitors or protease inhibitors; or triple nucleoside reverse-transcriptase inhibitors. TMP-SMX (80/400 mg) has been the recommended treatment and prophylaxis for pneumocystosis. For those patients who are unable to tolerate TMP-SMX, prophylaxis or treatment is replaced with clindamycin plus primaquine [14]. Atovaquone, pyrimethamine and dapsone are not available for pneumocystosis prophylaxis in Taiwan, while pentamidine was available only before 1997. The decision of discontinuation of prophylaxis or switch to clindamycin plus primaquine for pneumocystosis in the face of adverse effects of TMP-SMX has been made by the treating physicians.

### Follow-up and end points

After initiation of HAART, patients received follow-up at an interval of 1 to 3 months. Each visit included a medical history taking, a general physical examination, and laboratory tests including complete blood counts with differential count and blood biochemistry. CD4 count and PVL were determined before and 1 month after HAART and every 3-4 months thereafter. A chest radiograph and microbiological investigations were performed when patients presented with respiratory symptoms or a history of prolonged fevers or wasting.

Pneumocystosis was confirmed when cytology or histopathology of respiratory specimens disclosed cysts or trophozoites by special stains; pneumocystosis was considered presumptive if the patients presented with typical symptoms (resting or exertional dyspnea, nonproductive cough, hypoxemia, and elevated lactate dehydrogenase levels), plus radiographic manifestations of interstitial pneumonitis who responded to any of the standard recommended treatments for pneumocystosis.

Other than pneumocystosis, we also assessed the incidence of bacterial infection and parasitic infection after discontinuation of primary or secondary prophylaxis. A diagnosis of bacterial infection was made on microscopy and cultures of relevant clinical specimens and a compatible clinical history that included an acute onset of symptoms, with a response to anti-bacterials that have no known activity against pneumocystosis. Parasitic infections of interest were amebiasis, giardiasis, cryptosporidiosis, isoporiasis, cyclosporiasis, and toxoplasma encephalitis. The study was approved by Research Ethics Committee of the National Taiwan University Hospital on 25 July, 2001 (reference number: 19011). The study was conducted through periodic review of medical records retrospectively and was waived for informed consent.

### Statistical analysis

The observation duration for occurrence of pneumocystosis was estimated from the date of discontinuation of primary or secondary prophylaxis to the date of diagnosis of pneumocystosis, loss to follow-up at this hospital, or death, or the end of the study on 31 December, 2007, whichever occurred first. For patients who discontinued primary or secondary prophylaxis before the CD4 counts increased to 200 cells/μL (early discontinuation), the end date of observation was the mid-point between the last date when the patient's CD4 count was <200 cells/μL and the first date when the patient's CD4 count increased to ≧200 cells/μL.

All statistical analyses were performed using statistical software programs (SAS version 8.1; SAS Institute Inc, Cary, NC and STATA 10; College Station, Texas, USA). Categorical variables were expressed as proportions and compared using χ^2 ^by Fisher exact test (2-tailed). The incidence rate for each group was calculated as the number of cases of pneumocystosis, bacterial infection or parasitic infection per 100 PY of observation. Exact 95% confidence intervals (95% CI) for incidence rates were calculated on the basis of the Poisson distribution.

## Results

### Patient characteristics

Between April 1997 and September 2007, 660 patients fulfilled the inclusion criteria (Figure 1): 165 patients with baseline CD4 counts of <200 cells/μL did not receive primary prophylaxis for pneumocystosis; and 279 and 216 patients received primary and secondary prophylaxis, respectively. Their demographic and clinical characteristics at enrollment are shown in Table 1. Most patients were men, and the risk factor of HIV transmission was predominantly sexual contact.

**Table 1:** Characteristics of 495 patients who received prophylaxis and 165 patients who did not receive prophylaxis

Characteristics	Primary prophylaxis			Secondary prophylaxis			No prophylaxis
	**CD4 <200/μL**	**CD4 ≧200/μL**	**P value**	**CD4 <200/μL**	**CD4 ≧200/μL**	**P value**	
Patient number, n	211	68		145	71		165
Age at enrollment, mean (SD), y	39.0 (12.0)	35.0 (10.7)	0.03	38.6 (10.2)	35.0 (8.4)	0.006	37.0 (10.9)
Male sex, n (%)	195 (92.4)	66 (97.1)	0.26	134 (92.4)	68 (95.8)	0.56	152 (92.1)
Risk behaviors, no (%)							
MSM	138 (65.4)	48 (70.6)	0.74	89 (61.4)	58 (81.7)	0.22	110 (66.7)
Heterosexual	68 (32.2)	17 (25.0)	0.47	50 (34.5)	10 (14.1)	0.02	41 (24.8)
Others	5 (2.4)	3 (4.4)	0.41	6 (4.1)	3 (4.2)	0.99	14 (8.5)
Nadir CD4 count, median (range), cells/μL	27 (1-182)	93 (67-194)	<0.001	17 (0-165)	40 (1-174)	<0.001	134 (4-199)
Baseline PVL, median (range), log_10 _copies/ml	5.46 (2.60-6.12)	5.60 (2.60-6.19)	0.13	5.56 (2.60-5.99)	5.72 (3.09-5.98)	0.04	5.14 (2.60-5.88)
Chemoprophylaxis, n (%)							
Trimethoprim-sulfamethoxazole	207 (98.1)	67 (98.5)	1	142 (97.9)	71 (100)	1	NA
Clindamycin/primaquine	2 (0.9)	2 (2.9)	0.26	29 (20.0)	15 (21.1)	0.86	NA
Macrolides	16 (9.0)	5 (7.4)	1	6 (4.1)	7 (9.9)	0.14	NA
Duration of prophylaxis, median (range), m	2.0 (0.2-66.2)	5.3 (0.25-47.8)	0.036	2.7 (0.1-62)	4.0 (0.34-57)	0.04	
CD4 at discontinuation of prophylaxis							
median (range), cells/ul	101 (2-199)	234 (207-642)	<0.001	111 (5-197)	279 (201-880)	<0.001	
<100 cells/ul, n (%)	101 (47.9)	0 (0)		53 (36.6)	0 (0)		
PVL at discontinuation of prophylaxis, median (range), log_10 _copies/ml	2.60 (2.60-5.88)	2.60 (2.60-5.15)	0.027	2.60 (2.60-5.88)	2.60 (2.60-5.51)	0.022	NA
PVL < 400 copies/ml, no (%)	130 (61.6)	55 (80.9)	0.24	85 (58.6)	53 (74.6)	0.31	NA
Latest CD4, median (range), cells/ul	200 (2-214)	376 (0-952)	0.019	200 (2-215)	426 (2-1124)	0.005	200 (4-209)
<200 cells/ul, n (%)	78 (37.0)	15 (22)	0.11	41 (28.3)	10 (14.1)	0.09	49 (29.7)

Abbreviations: MSM. Men who have sex with men; NA, not applicable; PVL, plasma HIV RNA load

Among the 279 patients who received primary prophylaxis for pneumocystosis, 211 (75.6%) had early discontinuation of prophylaxis, and 68 (24.4%) continued prophylaxis until CD4 increased to ≧200 cells/μL (Table 1). Compared with the latter group of patients, the former group of patients were older (mean age, 39 vs 35 years; *P *= 0.03) and had a lower baseline CD4 count (median, 27 vs. 93 cells/μL; *P *< 0.001), while both groups of patients had similar baseline PVL (median, 5.46 vs 5.60 log_10 _copies/ml; *P *= 0.13). Forty-seven (16.8%) patients were lost to follow-up in this group.

Among the 216 patients who received secondary prophylaxis, 145 (67.1%) had early discontinuation of prophylaxis, and 71 (32.9%) continued prophylaxis until CD4 increased to ≧200 cells/μL (Table 1). Compared with the latter group of patients, the former group of patients were older (mean, 38.6 vs. 35 years; *P *= 0.006), had a lower baseline CD4 count (median, 17 vs. 40 cells/μL; *P *< 0.001) and had a lower baseline PVL (median, 5.56 vs 5.72 log_10 _copies/ml; *P *= 0.04) (Table 1). Twenty-three (10.6%) patients were lost to follow-up in this group.

Among the 165 patients who did not receive primary or secondary prophylaxis, the median baseline CD4 count was 134 cells/μL, and PVL was 5.14 log_10 _copies/ml (Table 1). Forty-four (26.6%) patients were lost to follow-up.

### Duration of prophylaxis and latest CD4 counts after HAART

The median duration of primary prophylaxis for pneumocystosis was significantly shorter for the 211 patients who had early discontinuation of prophylaxis than for the 68 patients who continued prophylaxis until CD4 count increased to ≧200 cells/μL (2.0 vs. 5.3 months). Although CD4 counts continued to increase with HAART (data not shown), the median CD4 count of patients who had early discontinuation of prophylaxis at the end of observation was significantly lower than that of the patients who continued prophylaxis until CD4 increased to ≧200 cells/μL (200 vs. 376 cells/μL) (table 1).

The median duration of secondary prophylaxis for pneumocystosis was significantly shorter for the 145 patients who had early discontinuation of prophylaxis than for the 71 patients who continued prophylaxis until CD4 increased to ≧200 cells/μL (2.7 vs. 4.0 months). The median CD4 count of the patients who had early discontinuation of prophylaxis at the end of observation was also lower than that of the patients who continued prophylaxis until CD4 increased to ≧200 cells/μL (200 vs. 426 cells/μL) (table 1).

Among the 165 patients who did not receive any prophylaxis, their median CD4 count at the end of observation was 200 cells/μL (4-209) after follow-up for 7.6 months while they continued HAART.

### Incidence of events during Follow-up

The crude incidence of adverse effects of primary prophylaxis with TMP-SMX was estimated 23.3%, which included skin rashes (13.3%), leucopenia (6.8%), gastrointestinal intolerance (2.9%), and Stevens-Johnson syndrome (0.4%). Among the 211 patients who had early discontinuation of primary prophylaxis, 11 cases of pneumocystosis were diagnosed after an observation duration of 366 PY (incidence rate, 3.0 per 100 PY; 95% CI, 1.50 to 5.38). These 11 episodes of presumptive diagnosis of pneumocystosis occurred in patients with a median CD4 count of 33 cells/μL (range, 8-138) after a median observation of 13.7 months (range, 2.2-92.4) in the absence of primary prophylaxis. Those episodes occurred in patients with frequent losses to follow-up for more than 1 year (9 patients) or poor compliance with HAART (2). Among the 68 patients who discontinued primary prophylaxis after CD4 counts increased to ≧200 cells/μL, 1 case of pneumocystosis was diagnosed with an incidence rate of 0.43 per 100 PY (95% CI, 0.01 to 2.38). In the meantime, 53 episodes of bacterial infections were diagnosed in patients who had early discontinuation of primary prophylaxis, yielding an incidence rate of 14.48 per 100 PY (95% CI, 10.85 to 18.94), while 13 episodes were diagnosed in the patients who discontinued primary prophylaxis after CD4 counts increased to ≧200 cells/μL, yielding an incidence rate of 5.56 per 100 PY (95% CI, 2.96 to 9.50) (*P *< 0.001). The types of bacterial infections of the former group included skin and soft tissue infections (34 cases), community-acquired pneumonia (7), urinary tract infection (5) and bacteremia due to methicillin-resistant *Staphylococcus aureus*, non-tuberculous mycobacteria or non-typhoid *Salmonella *(7). Two episodes of parasitic infection (toxoplasmosis), with an incidence rate of 0.54 per 100 PY (95% CI, 0.07 to 1.97), occurred in the patients who had early discontinuation of primary prophylaxis.

Forty-seven patients (21.8%) developed adverse effects that prompted discontinuation of secondary prophylaxis; those adverse effects included skin rashes (17.6%), gastrointestinal intolerance (2.3%) and leucopenia (1.9%). Among the 145 patients who had early discontinuation of secondary prophylaxis, 6 cases of pneumocystosis were diagnosed with an incidence rate of 2.49 per 100 PY (95% CI, 0.91 to 5.42) (Table 2). These 6 cases of presumptive pneumocystosis occurred in patients with a median CD4 count of 4 cells/μL (range, 1-40) after a median observation of 22.8 months (range, 8.3-69.3) in the absence of secondary prophylaxis. All of the episodes occurred in patients who had poor compliance with HAART and virologic failure. The incidence rate of pneumocystosis was 0.48 per 100 PY (95% CI, 0.01 to 2.65) in the 71 patients who discontinued secondary prophylaxis after CD4 counts increased to ≧200 cells/μL. In total, 21 episodes of bacterial infections were diagnosed in patients who had early discontinuation of secondary prophylaxis, yielding an incidence rate of 8.71 per 100 PY (95% CI, 5.39 to 13.32); and 7 episodes were diagnosed in patients who discontinued secondary prophylaxis after CD4 counts increased to ≧200 cells/μL, yielding an incidence rate of 3.33 per 100 PY (95% CI, 1.34 to 6.87). The types of bacterial infections in the former group included skin and soft tissue infections (13 cases), community-acquired pneumonia (4), urinary tract infection (1) and bacteremia due to *S. aureus *or non-typhoid *Salmonella *(3). Two episodes of parasitic infections (one amebiasis and one giardiasis), occurred in patients who had early discontinuation of secondary prophylaxis, yielding an incidence rate of 0.83 per 100 PY (95% CI, 0.10 to 3.00).

**Table 2:** Outcomes of patients who discontinued prophylaxis before and after cd4 count increase to ≧200 cells/μl and patients who did not initiated prophylaxis for pneumocystosis

Outcome	Primary prophylaxis			Secondary prophylaxis			**No prophylaxi**s
	**CD4 <200/μL**	**CD4 ≧200/μL**	**P value**	**CD4 <200/μL**	**CD4 ≧200/μL**	**P value**	
Patient number, n	211	68		145	71		165
New/recurrent episodes of PCP, n	11	1		6	1		3
Time from discontinuation of prophylaxis to PCP, median, M	13.7 (2.2-92.4)	74.5		22.8 (8.3-69.3)	-20.7		
CD4 count when new PCP developed, median, cells/μl	33 (8-138)	9		4 (1-40)	-13		5 (0-87)
Total observation duration, PY	366	234		241	210		104
Incidence of new/recurrent PCP, per 100 PY (95% CI)	3.00 (1.26-4.76)	0.43 (0.01-2.38)	0.03	2.49 (0.91-5.42)	0.48 (0.01-2.65)	0.13	2.88 (0.59-8.43)
Episodes of bacterial infection, n	53	13		21	7		22
Incidence of bacterial infection, per 100 PY (95% CI)	14.48 (10.85-18.94)	5.56 (2.96-9.50)	<0.001	8.71 (5.39-13.32)	3.33 (1.34-6.87)	0.03	21.16 (13.35-32.03)
Episodes of parasitic infection, n	2	0		2	0		2
Incidence of parasitic infection, per 100 PY (95% CI)	0.54 (0.07-1.97)	0		0.83 (0.10-3.00)	0		1.92 (0.23-6.95)
Death, n (%)	23 (10.9)	4 (5.9)	0.35	8 (5.5)	1 (1.4)	0.28	22 (13.3)
Patients lost to follow-up, n (%)	40 (19.0)	7 (10.3)	0.19	16 (11.0)	8 (11.3)	1	44 (26.6)

Abbreviations: CI, confidence interval; PCP, *Pneumocystis jirovecii *pneumonia; PY, persons-years

The incidence rate of pneumocystosis in 165 patients with baseline CD4 counts of <200 cells/μL who did not receive either primary or secondary prophylaxis for pneumocystosis was 2.88 per 100 PY (95% CI, 0.59 to 8.43) (Table 2). Twenty-two episodes of bacterial infections (incidence rate, 21.16 per 100 PY [95% CI, 13.35 to 32.03]) and two episodes of parasitic infections (1 amebiasis and 1 toxoplasmosis) (incidence rate, 1.92 per 100 PY [95% CI, 0.23 to 6.95]) were diagnosed. The types of bacterial infections included skin and soft tissue infections (13 cases), community-acquired pneumonia (3), urinary tract infection (2) and bacteremia due to *Escherichia coli *or non-typhoid *Salmonella *(4).

When patients who did not initiate prophylaxis or had early discontinuation of prophylaxis for pneumocystosis were analyzed together, 20 cases of pneumocystosis developed after a total observation duration of 711 PY, with an incidence rate of 2.81 per 100 PY (95% CI, 1.72, 4.34). Among the 139 patients who continued primary or secondary prophylaxis after CD4 counts increased to ≧200 cells/μL after HAART, 2 cases of pneumocystosis developed after a total observation duration of 444 PY, with an incidence rate of 0.45 per 100 PY (95% CI, 0.05, 1.63). The risk of pneumocystosis was significantly higher in the former group of patients than in the latter group, with a risk ratio of 5.32 (95% CI, 1.18, 23.94; *P *= 0.03) after adjustment for age, sex, risk for HIV transmission, and baseline CD4 and PVL.

Among the 215 patients who achieved undetectable PVL and had early discontinuation of primary of secondary prophylaxis for pneumocystosis, only one patient developed pneumocystosis after a total observation duration of 323 PY (incidence rate, 0.31 per 100 PY [95% CI, 0.01, 1.71]). The adjusted risk ratio for pneumocystosis was 0.63 (95% CI, 0.03, 14.89; *P *= 0.77) when the risk of these patients compared to that of the patients who discontinued primary or secondary prophylaxis after CD4 counts increased to ≧200 cells/μL.

## Discussion

In this study, we have found that, compared with the patients who continued primary or secondary prophylaxis for pneumocystosis until CD4 counts increased to ≧200 cells/μL after HAART, patients who had early discontinuation of prophylaxis were at a significantly higher risk for pneumocystosis, especially among those patients who did not adhere to HAART. In the subgroup analysis, the risk of pneumocystosis could be significantly reduced if the patients who had early discontinuation of prophylaxis complied with HAART and achieved undetectable PVL.

The incidence rate of pneumocystosis following discontinuation of primary and secondary prophylaxis among HIV-infected patients when their CD4 counts increased to ≧200 cells/μL after receiving HAART in published studies ranges from 0 to 2.27 cases per 100 PY of follow-up, depending on the types of study design and observation duration [3,11,15-25]. In our patients who followed the guidelines for discontinuation of pneumocystosis prophylaxis, the overall incidence was 0.45 per 100 PY (95% CI, 0.05, 1.63), which is within the range of reported incidence rates of case series and cohort studies (Table 3).

Discontinuation of prophylaxis before CD4 counts increase to ≧200 cells/μL after HAART has been performed in some selected patients in published observational studies [11,15,17]. In one study, primary or secondary prophylaxis was discontinued when CD4 count was <200 cells/μL in 98 patients who received HAART without occurrence of pneumocystosis [15]. In a recent study by D'Egidio et al. [11], no recurrence of pneumocystosis was demonstrated in 19 patients who had a median CD4 count of 120 cells/μL and achieved sustained HIV viral suppression before prophylaxis for pneumocystosis was discontinued during the mean follow-up duration of 13.7 months. The observations of increased overall risk for pneumocystosis in our patients with early discontinuation of prophylaxis and reduced risk for pneumocytosis in those patients who achieved undetectable PVL suggest that decision of earlier discontinuation of prophylaxis should only be cautiously made in selected patients who have a favorable immunologic or virologic response to HAART.

Earlier discontinuation of TMP-SMX or other alternatives in the patients with increasing CD4 counts may reduce pill burden, risk for adverse effects of TMP-SMX that was noted in one fourth of our patients, and risk for emergence of drug-resistant bacteria [26-28]. However, the benefit should be balanced against the risk because use of TMP-SMX is associated with decreased risk for bacterial infections [29,30]. In our study, the incidence rate of all-cause bacterial infection was estimated 8.71-21.16 per 100 PY of follow-up after discontinuation of prophylaxis for pneumocystosis, which appears to be higher than those of previous studies, which ranged from 1.99 to 12.21 per 100 PY of follow-up [19-22].

Our findings that most of the cases of pneumocystosis occurred in patients who did not have good adherence to HAART and HIV care should alert clinicians and patients with respect to the risk for pneumocystosis if decision of early discontinuation of prophylaxis has to be made in the face of adverse effects of antibacterial agents. For those who have to discontinue primary or secondary prophylaxis due to intolerance or adverse effects, adherence to HAART can not be overemphasized. Reinstitution of prophylaxis should be considered once immunologic failure occurs after virologic failure to HAART due to poor compliance and emergence of HIV that is antiretroviral-resistant.

There are several limitations of our study and interpretation of our study should be cautious. First, this is not a clinical trial to compare the risk for pneumocystosis between patients who discontinue primary or secondary prophylaxis when their CD4 counts remain < 200 cells/μL and those who discontinue prophylaxis when their CD4 counts increase to ≧200 cells/μL. Second, the decision of discontinuation of prophylaxis was made by treating physicians based on individualized assessment, which may be affected by uncontrolled factors. By medical record review, we were not able to know the reasons why the treating physicians discontinued prophylaxis earlier before CD4 increased to the recommended cut-off value by the guidelines. Third, the diagnosis of pneumocystosis was based on clinical suspicion and an appropriate response to any of the recommended standard treatments and the absence of evidence of bacterial pneumonia. All of our diagnoses were presumptive, not definitive. Fourth, the results of this study of early discontinuation of pneumocystosis prophylaxis may not be generalized to the patients with virologic and immunological failure or the patients with limited access to HAART and HIV care. Last, the benefits of TMP-SMX in resource-limited settings are multiple in addition to its low cost, and continuation of TMP-SMX can protect patients from several bacterial or parasitic infections [31-33]. In such settings, benefits of continuation of TMP-SMX may still outweigh the risks associated with earlier discontinuation of TMP-SMX after programs of antiretroviral therapy is increasingly implemented in these regions.

## Conclusions

The overall risk of pneumocystosis in patients on HAART was low in Taiwan. Patients who had early discontinuation of prophylaxis for pneumocystosis were associated with a higher risk of pneumocystosis and bacterial infections than those who continued prophylaxis until CD4 increased to ≧200 cells/μL after HAART. The risk of pneumocystosis was lower in those patients who had early discontinuation of prophylaxis while achieving complete virologic response to HAART.

## List of abbreviations

95% CI: 95%confidence interval; HAART: highly active antiretroviral therapy; HIV: human immunodeficiency virus; PVL: plasma HIV RNA load; 100 PY: 100 person-years; TMP-SMX: trimethoprim-sulfamethoxazole

## Competing interests

The authors declare that they have no competing interests.

## Authors' contributions

CYC, MYC, and CCH conceived of the study, and participated in its design and coordination. CYC, WHS, WCL, and CCH reviewed the medical records. CYC, WHS, HYS, YCL analyzed and interpreted the data. CYC, SMH, CCH drafted the manuscript. All authors read and approved the final manuscript.

## Pre-publication history

The pre-publication history for this paper can be accessed here:

http://www.biomedcentral.com/1471-2334/10/126/prepub
